# *Leishmaniavirus*-Dependent Metastatic Leishmaniasis Is Prevented by Blocking IL-17A

**DOI:** 10.1371/journal.ppat.1005852

**Published:** 2016-09-22

**Authors:** Mary-Anne Hartley, Eliane Bourreau, Matteo Rossi, Patrik Castiglioni, Remzi Onur Eren, Florence Prevel, Pierre Couppié, Suzanne M. Hickerson, Pascal Launois, Stephen M. Beverley, Catherine Ronet, Nicolas Fasel

**Affiliations:** 1 Department of Biochemistry, University of Lausanne, Epalinges, Switzerland; 2 Immunologie des Leishmanioses, Institut Pasteur de la Guyane, Cayenne, French Guiana; 3 Service de Dermatologie, Centre Hospitalier Andrée Rosemon, Cayenne, French Guiana; 4 Department of Molecular Microbiology, Washington University School of Medicine, Saint Louis, Missouri, United States of America; 5 World Health Organization Immunology Research and Training centre (WHO-IRTC), Epalinges, Switzerland; Imperial College London, UNITED KINGDOM

## Abstract

Cutaneous leishmaniasis has various outcomes, ranging from self-healing reddened papules to extensive open ulcerations that metastasise to secondary sites and are often resistant to standard therapies. In the case of *L*. *guyanensis* (*L*.*g*), about 5–10% of all infections result in metastatic complications. We recently showed that a cytoplasmic virus within *L*.*g* parasites (LRV1) is able to act as a potent innate immunogen, worsening disease outcome in a murine model. In this study, we investigated the immunophenotype of human patients infected by *L*.*g* and found a significant association between the inflammatory cytokine IL-17A, the presence of LRV1 and disease chronicity. Further, IL-17A was inversely correlated to the protective cytokine IFN-γ. These findings were experimentally corroborated in our murine model, where IL-17A produced in LRV1+ *L*.*g* infection contributed to parasite virulence and dissemination in the absence of IFN-γ. Additionally, IL-17A inhibition in mice using digoxin or SR1001, showed therapeutic promise in limiting parasite virulence. Thus, this murine model of LRV1-dependent infectious metastasis validated markers of disease chronicity in humans and elucidated the immunologic mechanism for the dissemination of *Leishmania* parasites to secondary sites. Moreover, it confirms the prognostic value of LRV1 and IL-17A detection to prevent metastatic leishmaniasis in human patients.

## Author Summary

Cutaneous leishmaniasis is a neglected parasitic disease particularly problematic in the South American tropics, where the parasites have an ability to migrate from the initial inoculation site in a process called “infectious metastasis”. Despite existing for centuries, the factors involved in parasite dissemination are largely unknown. We recently described that *Leishmaniavirus* (LRV), a virus present within some *Leishmania* parasites, is responsible for an exacerbation of the disease outcome in mice together with treatment failure and symptomatic relapse in human patients. In this paper we show that the expression of the pro-inflammatory cytokine IL-17A is correlated with disease chronicity in patients infected by LRV+ *Leishmania* parasites. We confirmed this finding in a unique murine model where IL-17A was also found to play a role in LRV1-dependent infectious metastasis. Additionally, using IL-17A as a prophylactic target, we were able to inhibit parasite proliferation and the development of infectious metastasis. Thus, we identify a pathogenic role for IL-17A in LRV1-dependent cutaneous leishmaniasis and infectious metastasis. More importantly, our data suggests that in addition to LRV presence, IL-17A is a marker of disease severity and could be a novel target to promote lesion resolution and prevent parasite dissemination.

## Introduction

Currently 5% of humanity lives at risk of *Leishmania* infection across 98 countries worldwide [1]. This threat is widening with climate change and vector spread [2] to include Southern Europe [3] and Northern America [4]. *Leishmania* parasites are mostly (80%) dermotropic, infesting and ulcerating human skin around the inoculation site of their haematophagous sand fly vector. While most infections remain localized at this site, several of the 20+ *Leishmania* species have a particular risk of infectious metastasis, where parasites migrate to unrelated cutaneous locations, causing secondary lesions that are often severely inflamed, deforming surrounding tissues into debilitating ulcerations, nodular granulomas or maculopapular rashes [5]. This may occur several years after resolution of the initial lesion where the extreme symptomatic diversity and poor predictability of the outcome poses major challenges to the diagnosis, treatment and control of metastatic leishmaniasis in the resource-scarce environments in which it is endemic [6, 7].

Naturally occurring *Leishmania*-RNA-viruses (LRV) of the *Totiviridae* family have been found in the cytoplasm of several parasite species [8–11]. In murine models, the dsRNA viral genome is recognized by host Toll-Like Receptor 3 (TLR3) [10, 12], initiating a potent innate anti-viral inflammatory cascade. In *L*.*g* infection, we have shown that this reaction exacerbates lesional inflammation and increases parasite survival in mice [12]. Furthermore, LRV1 was associated to treatment failure and mucosal manifestations in humans [13–15]. Interestingly, while the presence of LRV1 in *L*.*g* parasites is able to worsen the symptomatic outcome in our C57BL/6 murine model, the mice ultimately heal, with no evidence of secondary lesions [16]. Indeed, C57BL/6 mice are notorious for being resistant to numerous spp. of the *L*. *Leishmania* subgenus, such as *L*. *major*. Previously, it was shown that this resistance and susceptibility diverge with CD4+ T-cell polarisations whereby resistance is marked by a cell-mediated T_H_1 reaction, while mice succumb to disease under a T_H_2 polarisation [17, 18]. Since then, however, the true complexity of T-cell polarisation has been revealed, and the T_H_1/T_H_2 dichotomy in leishmaniasis has slowly shifted from a paradigm to a paradox [19]. For instance, CL in humans or disease models other than *L*. *major* have a much more graded and heterogeneous response, including potentially major roles for T_REG_, T_H_9 and T_H_17 populations [20–22]. Nevertheless, metastatic CL due to *L*. *guyanensis* is not documented in mice, and consequently, there is no simple model or information on the immunological determinants of leishmanial metastasis with which we could design immunotherapeutic interventions to prevent and treat this disfiguring clinical complication.

The role of the T_H_17 response is potentially noteworthy in hyper-inflammatory CL. Its key cytokine, IL-17A has a reputation as the architect of numerous chronic inflammatory diseases such as multiple sclerosis, inflammatory bowel disease, asthma, psoriasis, rheumatoid arthritis, contact dermatitis, systemic lupus erythrematous [23], several cancers [24] and even depression [25]. Amongst its various functions, IL-17A is well known to induce immune cell migration, recruitment and activation.

We had previously hypothesised LRV1 to be an inflammatory virulence factor leading to infectious chronicity and metastatic leishmaniasis in humans. To test this hypothesis, we investigated the inflammatory phenotype of human patients infected by LRV1+ *L*.*g* parasites, specifically in relation to the role of IL-17A and IFN-γ in chronic outcomes. These findings were validated in murine models in which we were also able to demonstrate that LRV1-dependent metastasis is mediated by IL-17A in a low or depleted IFN-γ environment. Finally, we demonstrate that blocking IL-17A expression is able to prevent LRV1-dependent pathology and thus that LRV1 and IL-17A have significant potential to guide prognosis and develop an immunotherapeutic approach for the treatment and prevention of this disfiguring form of the disease.

## Results

### IL-17A production is associated with LRV1 presence and disease chronicity in human cutaneous leishmaniasis

**Fig 1:**
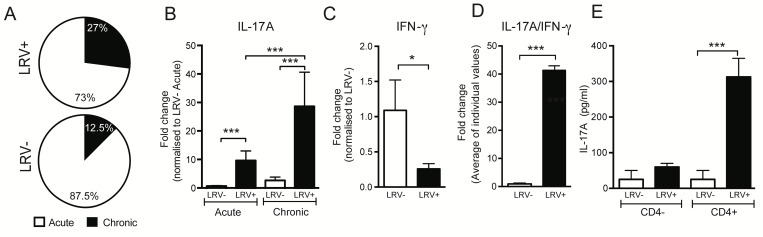
IL-17A production is associated with LRV1 presence and disease chronicity in human cutaneous leishmaniasis. 78 cutaneous leishmaniasis patients infected with *L*.*g* parasites were separated into two groups based on their LRV1 status (n = 30 LRV1+, n = 48 LRV1-). Two further clinical groups could be distinguished based on the duration of the patients’ symptomatic infection: 64 acute patients (lesion history of 0–5 months) and 14 chronic patients (lesion history of 5–12 months). **(A)** Distribution of symptomatic cohorts among LRV1+ and LRV1- infection. **(B)** IL-17A transcripts were measured in all biopsies by qRT-PCR. Values were calculated using the ∆∆CT method and normalized to the average of LRV1-*L*.*g* infection in the acute cohort. **(C)** IFN-γ transcripts were measured in biopsies of acute patients by qRT-PCR and normalized as before. **(D)** Previous qRT-PCR results were expressed for each individual as an IL-17A/IFN-γ ratio. **(E)** Lymphocytes were extracted from blood samples of acute patients (LRV+ n = 5, LRV- n = 4) and separated into CD4+ and CD4- groups by MACS. Cells were re-stimulated *ex vivo* with *L*.*g* parasites prior to IL-17A quantification by ELISA in supernatants. Data are mean +/- SEM using at least 2 technical replicates per condition. Significance tested by an unpaired, parametric *t*-test and indicated as *: P<0.05, **P<0.005, ***P<0.0001.

To establish the clinical relevance of IL-17A in LRV1+ *L*.*g* infections, we screened 78 adult human CL patients infected by *L*.*g* parasites in French Guiana. Patients were diagnosed with leishmaniasis and separated into two groups depending on the presence of LRV1. In this investigation, 30 LRV1+ *L*.*g* and 48 LRV1- *L*.*g* infected adults were studied. Selection criteria for this cohort included HIV-negative patients who were otherwise healthy and who had not previously received anti-leishmanial treatment. Among the 78 selected patients, 14 (18%) presented with chronic lesions that had persisted for over 5 months, whereas the remaining patients (82%) were enrolled during the acute phase of their disease. Patients infected by LRV1+ *L*.*g* parasites had the largest proportion of chronic lesions (8/30 patients, 27%), which was more than double of that found in LRV1*- L*.*g* infections (6/48 patients, 12.5%) **(Fig 1A)**. Due to the limited group size, this observation could not be reliably tested for statistical significance.

In order to identify patterns in IL-17A secretion between acute and chronic outcomes, a qRT-PCR was performed on tissue from intralesional biopsies. Analysis showed a significant correlation between IL-17A and the presence of LRV1 as well as the outcome of chronicity **(Fig 1B)**. Further, immunophenotyping in patients presenting with acute disease revealed that LRV1-associated IL-17A secretion was inversely related to IFN-γ secretion **(Fig 1C)**, suggesting that LRV1 detection is predictive of inflammatory responses involving a high IL-17A/IFN-γ ratio **(Fig 1D)**.

Patient blood samples provided a larger number of lymphocytes with which we could determine the identity of IL-17A-producing cells and confirm IL-17A production at the protein level. To this end, lymphocytes isolated from blood samples were re-stimulated *ex vivo* with live *L*.*g* parasites and an ELISA was performed on the cell-free supernatant. Similarly to the intralesional biopsies, samples from the LRV1+ cohort had significantly increased levels of IL-17A compared to their LRV1- counterparts (**S1 Fig**). Analysing the IL-17A contributions of the CD4+ and CD4- cell populations, we found that CD4+ T cells produced the vast majority (~80%) of LRV1-induced IL-17A in human patients **(Fig 1E)**.

### LRV1 induces IL-17A secretion in murine leishmaniasis and contributes to LRV1-mediated disease severity

**Fig 2:**
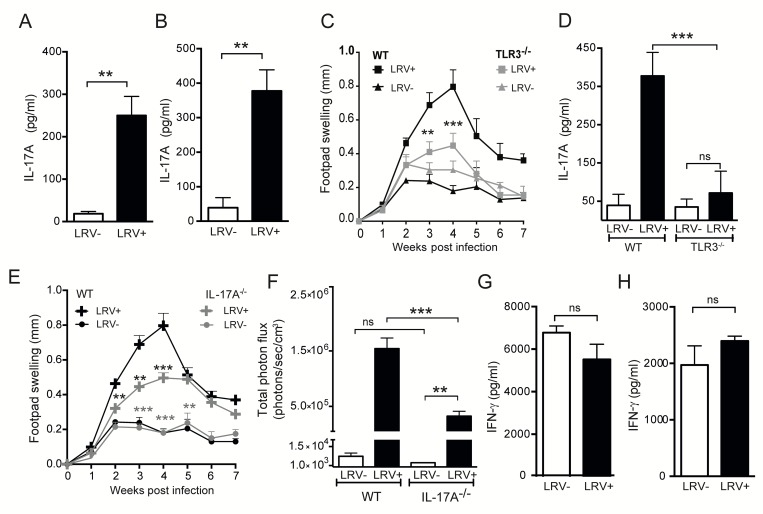
LRV1 induces IL-17A secretion in murine leishmaniasis and contributes to LRV1-mediated disease severity. Mice deficient in IL-17A or TLR3 and their WT controls (C57BL/6) were infected in the hind footpads with 3x10^6^ of either LRV1+ or LRV1-*L*.*g* stationary-phase promastigotes. At the peak of infection (4 weeks post-inoculation), cells from the footpad lesions and popliteal LNs were re-stimulated *ex vivo*. IL-17A secretion in WT mice was quantified in the supernatant by ELISA in **(A)** intra-lesional biopsies and **(B)** popliteal LNs. **(C)** Change in footpad swelling in TLR3^-/-^ and WT mice was measured weekly as a proxy for disease score. **(D)** IL-17A secretion from popliteal LN cells was also compared between WT and TLR3^-/-^ mice. **(E)** Change in footpad swelling in IL-17A^-/-^ and WT mice was measured weekly as a proxy for disease score. Statistical analysis is indicated in black for comparison between WT and IL-17A^-/-^ LRV1+ infected mice, and in grey for comparison between IL-17A^-/-^ LRV+ and LRV- infected mice. **(F)** At the peak of infection (week 4), the parasite burden of the mice depicted in **(E)** was quantified by *in vivo* parasite luminescence, after injecting mice intra-peritoneally with luciferin. As previously for IL-17A, IFN-γ secretion in WT mice was quantified in the supernatant of restimulated cells by ELISA in **(G)** intra-lesional biopsies and **(H)** popliteal LNs. Graphs are representative of a minimum of 3 independent experiments, using at least 5 mice per condition and presented as mean ± SEM. Significance tested by an unpaired, parametric *t*-test (bar graphs) or one-way Anova (disease score), and indicated as *: P<0.05, **P<0.005, ***P<0.0001.

To validate whether IL-17A could be predictive of chronic inflammation, we inoculated mice in the hind footpads with LRV1+ or LRV1-*L*.*g* parasites. At the peak of infection, lymphocytes were isolated from lesional tissue and draining popliteal lymph nodes (LNs) and then re-stimulated *ex vivo* with UV irradiated *L*.*g* parasites. Similarly to the human cohort, ELISA showed a ~10-fold LRV1-dependent increase of IL-17A in both lesional biopsies **(Fig 2A)** and draining LNs **(Fig 2B)**, which was abrogated in TLR3^-/-^ mice **(Fig 2D)**. Further analysis indicated that the quantity of IL-17A (rather than its source) dictated LRV1 pathology **(S2 Fig)**. To explore the relevance of LRV1-dependent IL-17A production, we then infected IL-17A-deficient mice with LRV1+ or LRV1-*L*.*g* parasites and found that they had a significantly reduced LRV1-mediated lesional swelling **(Fig 2E)** but to a lesser extent than seen in TLR3^-/-^ mice **(Fig 2C)**. IL-17^-/-^ mice also showed a reduced parasite burden at the peak of infection **(Fig 2F),** thus inferring a detrimental role for LRV1-dependent IL-17A and confirming the existence of a pathogenic TLR3-IL-17A signalling axis [26–30]. Importantly, a significant difference in lesion size **(Fig 2E)** and parasite growth **(Fig 2F)**, is visible between LRV- and LRV+ strains within the IL-17A^-/-^ mice, thus revealing that IL-17A is only one element of the complex pathogenesis of LRV1.

In contrast to the human samples, the level of the protective cytokine, IFN-γ, was not significantly different between LRV1+ and LRV1-*L*.*g* infections in this self-healing murine model of disease (for lesions **Fig 2G**, and LNs **Fig 2H**).

### LRV1-dependent infectious metastasis occurs in the absence of IFN-γ

**Fig 3:**
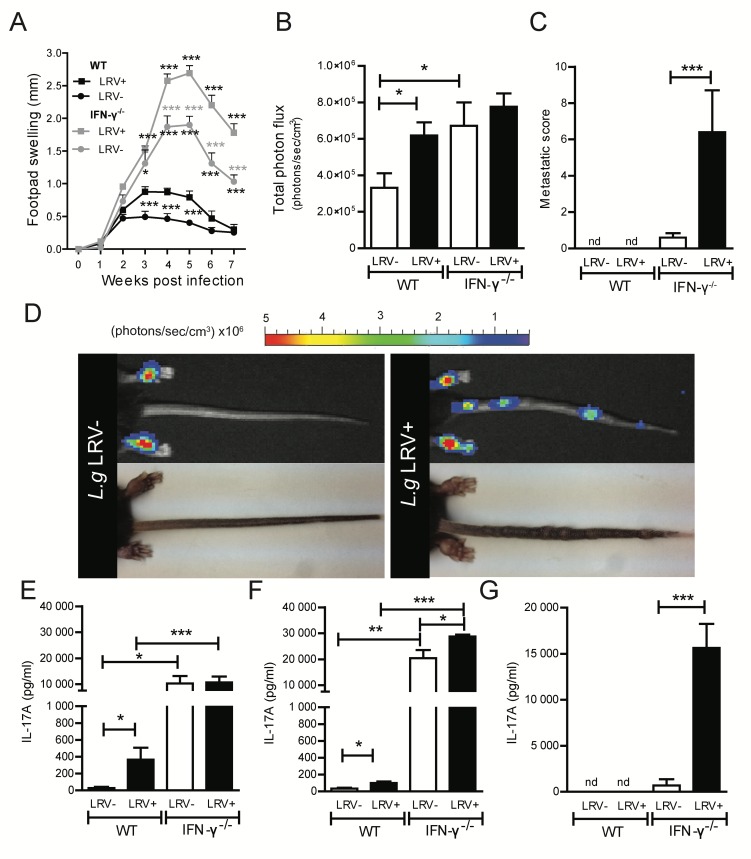
LRV1-dependent infectious metastasis occurs in the absence of IFN-γ. Mice deficient in IFN-γ and their WT controls (C57BL/6) were infected in the hind footpads with LRV1+ or LRV1-*L*.*g* stationary-phase promastigotes. **(A)** The change in footpad swelling was measured weekly as a proxy for disease progression. Statistical analysis is indicated for comparisons between WT LRV1+ infected mice in black and IFN-γ^-/-^ mice in grey. **(B)** At the peak of WT infection (week 4) *in vivo* parasite luminescence was determined as a measure of parasite burden. **(D)** At 7 weeks of infection, LRV1+*L*.*g* infected IFN-γ^-/-^ mice developed secondary lesions in their tails which were positive for parasite luminescence. **(C)** The number of secondary lesions was counted in all groups of mice. **(E)** At 4 weeks post infection, lymphocytes were extracted from popliteal LNs draining the primary lesion and then re-stimulated *ex vivo*. IL-17A secretion was quantified by ELISA in cell-free supernatants. **(F-G)** At 7 weeks post infection, lymphocytes were extracted from **(F)** popliteal LNs and **(F)** iliac LNs draining metastatic lesions, and restimulated. IL-17A production was quantified by ELISA as above. Graphs are representative of a minimum of 3 independent experiments, using at least 5 mice per condition and presented as mean ± SEM. Significance tested by an unpaired, parametric *t*-test, (bar graphs) or one-way Anova (disease score), and indicated as *: P<0.05, **P<0.005, ***P<0.0001.

To mimic the low IFN-γ levels seen in patients with LRV1+*L*.*g* infection, we tested whether we could establish chronicity and metastasis in IFN-γ-deficient mice. As expected, both LRV1+ and LRV1-*L*.*g* parasites were able to establish chronic disease in the absence of IFN-γ, however, between weeks 4–6, LRV1+*L*.*g* infection maintained a significantly higher pathology and parasite burden compared to its LRV1-*L*.*g* counterpart in IFN-γ^-/-^ mice **(Fig 3A and 3B)**. Interestingly, IFN-γ^-/-^ mice infected with LRV1+*L*.*g* developed multiple visible metastases on the tail (and occasionally the fore-paws/snout) with highly significant differences in the number of metastases between LRV1+ and LRV1- infections **(Fig 3C)**. Eventually, LRV1-*L*.*g* infected mice also developed metastases to a similar level of their LRV1+ counterparts but only after an additional 3–4 weeks of infection **(S3 Fig)**. All metastases tested positively for parasite-specific luciferase activity **(Fig 3D).** Taken together, these results illustrated that metastasis is a phenomenon occurring in the absence of IFN-γ and is significantly accelerated by the presence LRV1.

### LRV1-dependent IL-17A secretion is exaggerated in the absence of IFN-γ

It has been reported that IFN-γ signalling is inhibitory to IL-17A secretion [31–34]. Investigating LRV1-mediated IL-17A secretion in the popliteal LNs draining the primary lesions, we found that IL-17A was significantly increased in the absence of IFN-γ and exaggerated in the presence in LRV1 **(Fig 3E)**. Further, in IFN-γ^-/-^ mice infected with LRV1+*L*.*g* parasites, significant induction of IL-17A was found in LNs draining the footpad **(Fig 3F)** and metastatic tail lesions **(Fig 3G)**.

### IL-17A mediates LRV1-dependent metastasis in the absence of IFN-γ

**Fig 4:**
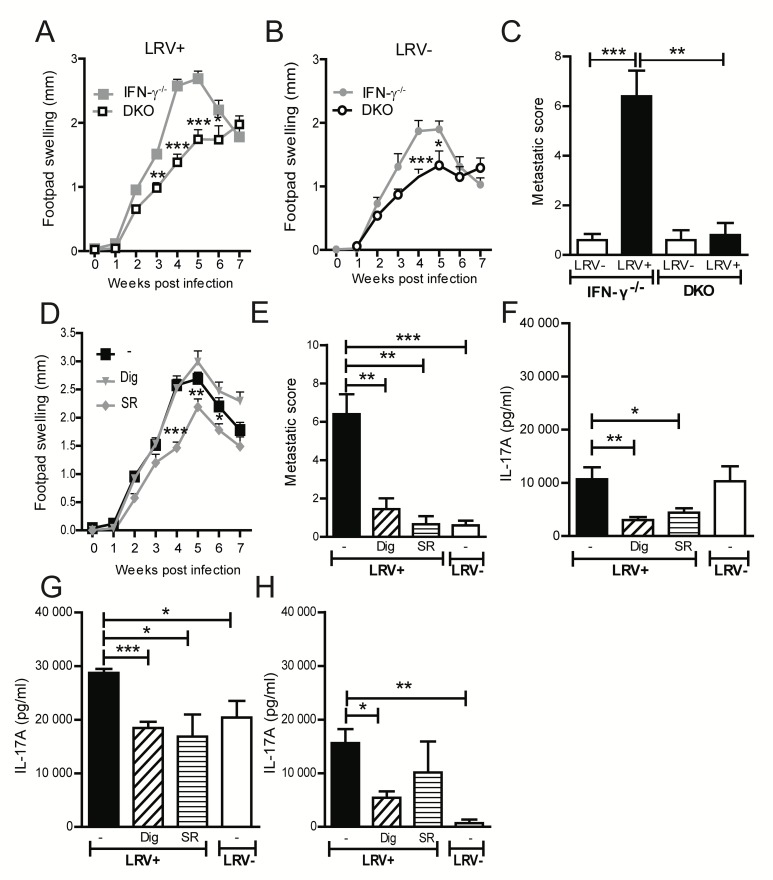
IL-17A mediates LRV1-dependent metastasis. **(A-C)** Mice deficient in IFN-γ^-/-^ or IFN-γ^-/-^ IL-17A^-/-^ (DKO) were infected in footpads with either **(A)** LRV1+ or **(B)** LRV1- *L*.*g* promastigotes. Footpad swelling was measured weekly. **(C)** The number of secondary lesions was counted in all groups at 7 weeks post infection. **(D-H)** IFN-γ^-/-^ mice were infected in their footpads with LRV1+/- *L*.*g* parasites. At the onset of visible lesions (2.5 weeks post infection) IFN-γ^-/-^ mice were treated i.p with either one of the IL-17A inhibitors every 2 days, namely Digoxin (Dig) or SR1001 (SR). **(D)** The change in footpad swelling was measured weekly. **(E)** At 7 weeks post infection, the number of secondary lesions was counted in all groups of treated mice. **(F)** At week 4 post infection, popliteal lymph node cells from mice presented in **(D)**, were restimulated for 72h *in vitro* with UV inactivated *L*.*g* parasites. IL-17A production in culture supernatants was measured by ELISA. After 7 weeks, lymphocytes from **(G)** popliteal and **(H)** iliac lymph nodes were also restimulated *in vitro* in order to quantify IL-17A production by ELISA. Graphs are representative of a minimum of 2 independent experiments, using at least 5 mice per condition and represented as mean ± SEM. Significance is tested by one-way Anova (disease score) or an unpaired, parametric *t*-test (bar graphs) and indicated as *: P<0.05, **P<0.005, ***P<0.0001.

Our human data suggested a direct correlation between IL-17A production, LRV1 presence and lesion chronicity (inversely related to IFN-γ production) **(Fig 1).** We thus set out to determine the role of IL-17A in LRV1-mediated infectious metastasis in a murine model of disease. To this end, we generated IFN-γ^-/-^IL-17A^-/-^ double knockout mice (DKO) with which we could examine IL-17A function in IFN-γ deficient environments. We confirmed the relevance of IL-17A by infecting DKO mice. Although we could observe reduced infection with either LRV1+ (**Fig 4A**) or LRV-*L*.*g* parasites (**Fig 4B**) compared to IFN-γ ^-/-^ mice, the DKO animals rarely developed metastasis by week 7 (**Fig 4C**)**.** These results suggest that blocking IL-17A expression could prevent LRV1-parasite dissemination to secondary lesions.

### IL-17A inhibiting drugs, digoxin and SR1001, reduce LRV1-mediated disease severity in mice

We subsequently tested whether digoxin (shown to inhibit IL-17A production [35] and possibly tumour metastasis [36]) and SR1001 (described to impede the differentiation and function of T_H_17 cells [35, 37]) could reduce LRV1 mediated disease severity in our murine model. Digoxin and SR1001 treatments were commenced at the onset of visible lesional swelling (2.5 weeks post infection). The drugs were delivered systemically via intra-peritoneal injections every second day, at concentrations previously reported to reduce IL-17A secretion *in vivo*. In LRV1+*L*.*g* infected C57BL/6 mice, we found that both drugs had an effect on lesional swelling **(S4A Fig)** and reduced parasite burden to levels found in IL-17A^-/-^ mice **(S4D Fig).** IL-17A secretion in draining popliteal LNs was also decreased **(S4C Fig).** Additionally, as expected, there was no effect in IL-17A^-/-^ mice **(S4D and S4F Fig)**. We then repeated this experiment in IFN-γ^-/-^ mice **(Fig 4D).** When lesions started to appear (week 2.5), digoxin and SR1001 were injected i.p. Although we didn’t observe a major effect on footpad swelling, such treatments significantly blocked the development of secondary lesions in the tail **(Fig 4E)** and decreased IL-17A secretion in the popliteal nodes at 4 weeks post infection **(Fig 4F),** and in the popliteal and iliac lymph nodes 7 weeks post infection **(Fig 4G–4H)**.

## Discussion

*L*. *guyanensis* is one of the major parasite species plaguing populations of the Amazonian basin. When present, its endosymbiotic virus (LRV1) acts as a potent innate immunogen capable of worsening lesional swelling in mice [12] and is correlated with first-line treatment failure, symptomatic relapse and metastasis in humans [13, 15]. Similarly, LRV1 presence in *L*. *braziliensis* was linked to treatment failure in humans [14] and exacerbated pathology in an HIV-*L*. *braziliensis* co-infected patient [38]. Thus far, epidemiological evidence suggests that LRV1 prevalence is highly variable among *Leishmania* species and is concentrated in certain geographic locations [13, 39, 40]. While LRV1 is present in some major metastatic strains of *Leishmania* and has been linked to metastasis in humans [15], no large-scale epidemiological data exist to assess the true correlation between LRV presence and metastatic leishmaniasis. For instance, LRV has not yet been detected in the metastatic strains of *L*. *panamensis* and a small-scale study has shown that metastasis is not exclusively associated with LRV presence in *L*. *braziliensis* [41]. Thus suggesting that infectious metastasis is a complex multifactorial process, in which parasite phylogeny, host immunocompetence, co-infecting pathogens, such helminths or viruses [42, 43] and environmental influences may also play a role [5].

Little is known about the immunology of *L*.*g* infections and their metastatic behaviour as few experimental studies have been performed on these parasites. While the presence of LRV1 is able to induce visible disease in C57BL/6 mice, it is nevertheless self-healing, where swelling and parasite load dissipates over the course of 7 weeks [12]. The innate resistance of C57BL/6 mice to most *Leishmania* strains is often attributed to a T_H_1 bias, in which abundant IFN-γ is able to activate a parasitotoxic respiratory burst in macrophages. Equally, however, excessive T_H_1 signalling has been blamed for the destructive inflammatory effects in some chronic leishmaniases [44–46]. Thus, many reports have described T_H_1 to have a “window of protective opportunity”, being damaging in excess and permissive to parasite proliferation in absence (reviewed in [47]). Our study on human patients found that LRV1+ *L*.*g* infections had a significantly reduced level of IFN-γ compared to LRV1- *L*.*g*. Interestingly, the opposite trend was found with the inflammatory cytokine, IL-17A, indicating that LRV1 detection could be predictive for this inflammatory imbalance in human CL. Supporting this hypothesis was that both LRV1 and IL-17A significantly correlated to lesion chronicity. We were able to validate these observations in a C57BL/6 murine model of disease, where we also found that IL-17A contributed to LRV1-mediated pathology and parasite survival. A significant observation in the IL-17A^-/-^ murine model was that LRV1-mediated pathology was not completely abrogated in the absence of IL-17A, thus demonstrating that IL-17A is only part of the complex pathogenic inflammatory response induced by LRV.

Indeed, a recent publication showed that IL-17A worsened CL caused by *L*. *major* in a susceptible BALB/c murine model that is notorious for its reduced level of IFN-γ [48]. This finding could also be reproduced in *L*. *major* infected C57BL/6 mice but only in the absence of IL-10 and especially in conditions of reduced IFN-γ signalling [49]. The destructive potential of IL-17A in leishmaniasis has also been corroborated in human disease, where IL-17A-mediated cell infiltration was linked with tissue damage in human mucosal leishmaniasis caused by *L*. *braziliensis* parasites [50]. The variable roles of IL-17A across murine models might be linked to differences in the type and potency of concomitant IFN-γ signalling. Interestingly, in the murine models of *L*. *braziliensis* and *L*. *amazonensis*, the infections were linked with IL-17A-producing T-cells. However, progressive *L*. *amazonensis* did so in an IFN-γ low environment [51] while submissive *L*. *braziliensis* parasites were associated with IFN-γ high CD4+ T cells [52], thus indicating that IFN-γ might control the pathogenicity of IL-17A in Neotropical leishmaniases. Unfortunately, however, these cytokines were not tested for their role in infectious metastasis and the LRV-status of the parasites used in the above studies remains unknown.

Our observation that IFN-γ levels are suppressed during LRV1+ *L*.*g* infection in human patients may corroborate our previous finding where suppressive regulatory T cells were demonstrated to accumulate in CL lesions of the Neotropics, leading to a suppression of IFN-γ and exacerbated disease outcome [53]. As IFN-γ levels were unchanged between LRV1+ and LRV1- *L*.*g* infections in our C57BL/6 murine model, we hypothesised that IFN-γ was responsible for disease resolution. Indeed, infection in an IFN-γ^-/-^ mouse resulted in chronic disease in both LRV1+ and LRV1- *L*.*g* infections. However, only IFN-γ^-/-^ mice infected with LRV1+ *L*.*g* parasites developed secondary lesions at 7 weeks post infection. On the other hand, LRV1- *L*.*g* infection required an additional 3–4 weeks to develop the same level of metastatic disease. Thus, this is the first study to describe that LRV1 is associated with accelerated infectious metastasis in murine cutaneous leishmaniasis. The pattern of metastasis in this murine model was anatomically predictable, appearing 95% of the time in the experimentally accessible and easily visible cutaneous tissue of the tail (occasionally, lesions were also found on the forepaw or snout). A common feature of these regions is the slightly cooler skin temperatures and fewer hair follicles, allowing us to speculate that either the parasites seek out cooler skin regions, or that lesions occur in regions frequently exposed to physical irritation such as is observed in humans [54], and in an experimental murine model of distal skin trauma [55].

In an effort to screen for immunological determinants of metastatic leishmaniasis, we used the IFN-γ^-/-^ mouse as a model of infectious metastasis. Similarly to the inverse correlation we observed in the human cohort, IL-17A secretion was exaggerated in the absence of IFN-γ. Further, removing IL-17A from our metastatic model significantly delayed the incidence of infectious metastasis. While it is clear that IL-17A is secreted in response to a variety of *Leishmania* species, the molecules triggering this response have not yet been described. The Paleotropic *L*. *major* species is generally LRV-negative (with one exception [56]). Considering that IL-17A has been shown to enhance disease severity in these assumedly LRV-negative *L*. *major* infections (in both BALB/c [48] and C57BL/6 [49] mice), there are certainly LRV1-independent triggers of IL-17A secretion. In our model, IL-17A secretion is also detected in lesions and draining LNs of LRV1- *L*.*g* infection but is significantly up-regulated in response to LRV1. Thus, we expect that an IL-17A amplification mechanism exists downstream from the LRV1-induced anti-viral signalling pathway of TLR3 [12]. Interestingly, previous reports support the existence of a TLR3-IL-17A signalling axis [26, 28]. The connection between TLR3 and IL-17A could also rely on the micro-RNA (miR)-155 [57]. In fact, miR-155 is thought to be essential for the development and maintenance of T_H_17 cells, where miR-155 interacts with T_H_17 transcription factors, STAT3 [58] and SOCS1 [59]. Further, miR-155^-/-^ T_H_17 cells are hypo-responsive to a key maintenance cytokine, IL-23 [60]. Together, these observations suggest that miR-155 could play an important role in IL-17A-related pathology in CL and deserves further investigation.

A wide variety of cells are able to produce IL-17A. Our analysis of human blood samples revealed the vast majority of LRV1-associated IL-17A to be produced by CD4+ T cells. However, further investigation in our murine model revealed the source of IL-17A to be more heterogeneous. Irrespective of the LRV-status of the infecting parasite, the majority of IL-17A-producing cells were CD3+ T cells carrying the classic TCRαβ: CD4+, CD8+ and also T cells double negative (DN) for CD4 and CD8. The former cell type has been recently appreciated as an important determinant of pathology in CL [61], where they have been described as hyper-activated inflammatory cells [62]. Further, double negative T cells are already well known for their ability to produce pathological IL-17A in the autoimmune disease systemic lupus erythrematous [63]. IL-17A was also produced by a significant proportion of CD3+ T cells hosting TCRγδ, however, testing in a deficient mouse model ruled out this population as a determinant of LRV1-mediated pathology **(S5 Fig)**.

Having established the pathogenic role of IL-17A in LRV1-mediated pathology, we explored whether depleting the cytokine in our murine model could have therapeutic benefits. While IL-17A neutralizing antibodies are available, this is not an economically feasible treatment option for the vast majority of leishmaniasis patients. Thus, we explored less expensive IL-17A blocking options, namely digoxin and its derivative, SR1001 which are able to inhibit the differentiation and functioning of T_H_17 cells by inducing inhibitory conformational changes in two key IL-17A transcription factors, RORα and RORγt [35, 37]. It was previously shown that daily intra-peritoneal doses of these drugs were able to reduce the clinical severity of a murine model of IL-17A-mediated experimental autoimmune encephalomyelitis (EAE) [35, 37]. Similarly, by treating our C57BL/6 murine model of leishmaniasis with digoxin and SR1001, we were able to significantly decrease IL-17A production in draining LNs resulting in a reduction of disease severity that reached levels comparable to that of the IL-17A^-/-^ model. Interestingly similar treatment in IFN-γ^-/-^ mouse demonstrated a crucial role for IL-17A downstream pathways in parasite dissemination to secondary lesions. Thus far, our testing in IL-17A^-/-^ and IFN-γ^-/-^ IL-17A^-/-^ mice showed no additional effects of digoxin or SR1001 on parasite burden, indicating that both drugs do not have IL-17A independent parasitotoxic activities *in vivo*. Further, as IL-17A has been shown to be pathologic in other forms of leishmaniasis, these drugs may hold therapeutic potential across a broader range of leishmanial species.

In conclusion, this report describes the first murine model of LRV1-mediated cutaneous leishmanial metastasis. We show that this process is mediated by IL-17A in the absence of IFN-γ and that IL-17A-inhibiting drugs are able to reduce LRV1-associated pathology. These results confirm our observations in a human cohort, where IL-17A production was linked to both the presence of LRV1 and disease chronicity.

Taken together, these results indicate that the detection of LRV1 or IL-17A in leishmaniasis has significant prognostic value and could be used to guide the therapeutic approach for the prevention and treatment of metastatic leishmaniasis.

## Methods

### Human patient selection and sample collection

Patients were received at the Centre Hospitalier Andrée Rosemon in Cayenne, French Guiana. Inclusion criteria selected newly diagnosed adult patients infected with *L*.*g* who had never previously received anti-leishmanials. Lesional history ranged from 1-12 months. HIV testing was conducted on an opt-in basis, and was a requirement for pre-selection, where only HIV-negative patients were included in order to maintain immunocompetence within the cohort. Similarly, a patient history revealing other autoimmune or infectious diseases was considered an exclusion criterion. Samples collected included: serous dermal fluid from the lesional border (lesional exudate), blood and a 2 mm intra-lesional punch biopsy. In total, 78 patient samples fitted these criteria (30 LRV1+ and 48 LRV1-).

### Leishmaniasis diagnostics and parasite phenotyping

Following local guidelines and standard protocol, diagnosis of leishmaniasis was based on microscopic analysis of lesional exudate by May-Grünwald Giemsa staining. Microscopy results were confirmed by *in vitro* parasite culture, where Schneider’s culture medium (described below) was inoculated with a segment of the 2 mm intra-lesional punch biopsy. Parasite cultures could then be typed by phylogenic RFLP-PCR analysis (based on a conserved isoenzyme polymorphism as previously described [64]).

#### *In vitro* parasite culture

The two isogenic clones of *L*.*g* infected with, or depleted of LRV1 (termed LRV1+ or LRV1- respectively), used in this study were described and characterized previously [65]. Briefly, these lines were derived from the LRV1+ parent strain, *L*.*g* M4147 (MHOM/BR/75/M4147) or an LRV1-null derivative [66]. The parasites express similar levels of a firefly luciferase (5x10^7^ photons/sec/10^6^ parasites) from a LUC gene integrated stably into the small subunit gene of the ribosomal RNA locus.

Parasites were cultured *in vitro* as promastigotes at 26°C in freshly prepared Schneider’s insect medium (Sigma) supplemented with 10% heat-inactivated foetal bovine serum (PAA), 10mM HEPES and 50U/ml penicillin/streptomycin (Animed), 0.6 mg/L biopterin and 5 mg/L hemin (Sigma-Aldrich). Each passage yielded infectious metacyclic promastigotes after 6 days and stocks were kept for no longer than 5 passages.

#### LRV1 detection in parasite isolates and biopsies

Parasite cultures were tested for LRV1 by qRT-PCR performed on both parasite cultures and lesional material using primers specific to a conserved LRV1 sequence (listed below). Briefly, total RNA was isolated using the RNeasy minikit (Qiagen) as previously described [67]. cDNA synthesis was performed on total RNA by using a first-strand cDNA synthesis kit (Superscript III, Invitrogen). qRT-PCR was carried out on a 7300 Real Time System (Applied Biosystems). To verify results and control for RNA contamination, parasite cultures were randomly selected and re-tested by various immuno-detection techniques as previously described [11]. Briefly, an antibody recognizing dsRNA irrespective of its underlying sequence (J2- English & Scientific Consulting) was used for dot-blot, ELISA and immunofluorescence detection. LRV1 presence was confirmed relative to the LRV1+ *L*.*g* (MHOM/BR/75/M4147) and the LRV1- *L*. *panamensis* (MHOM/PA/1971/LS94) parasites, used respectively as positive and negative controls.

### Cytokine quantification in human lesion biopsies and blood samples

#### Lesional tissue

Total RNA was isolated from lesional tissue similarly to the LRV1 qRT-PCR above. qRT-PCR was then performed on cDNA, testing for *il17a* and *ifn* **γ** transcripts (primers listed below).

#### Blood

Leukocytes were isolated from human blood in a Ficoll (Sigma-Aldrich) density gradient according to manufacturer’s instruction. A part of these cells were separated into CD4+ and CD4- compartments by magnet-activated cell sorting (MACs) using a positive selection CD4+ cell kit (Miltenyi Biotec). Selected cells were re-stimulated at a concentration of 1x10^6^ cells/ml for 5 days in RPMI. To these sorted blood cells, a mixture of LRV+ *L*.*g* parasites (1x10^6^/ml) and antigen presenting cells (1x10^5^/ml,) was added and incubated during 5 days at 37°C. Antigen presenting cells were autologous PBMCs (treated with 1μg/ml of mitomycin C during 30 min at 37°C).

Cell-free supernatants were then submitted to ELISA testing for IL-17A and IFN-γ quantification (eBioscience).

### Mouse facility, breeding and infection

All mice were bred in a specific pathogen-free housing facility at the University of Lausanne, Switzerland and backcrossed at least 10 times onto a C57BL/6 genetic background (purchased from Harlan Laboratories, Netherlands). The IFN-γ^-/-^ strain was purchased from Jackson laboratories, while IL-17A^-/-^ mice were generated by Prof. Y. Iwakura at the University of Tokyo, Japan [68]. These two strains were crossed to make a double knockout of IFN-γ ^-/-^IL-17A^-/-^ (DKO).

Age-matched (6–8 week old) female mice were infected in the hind footpads with 3x10^6^ stationary phase *L*.*g* promastigotes from either the LRV1+ or LRV1- strains. Change in footpad swelling was measured weekly using a Vernier caliper as a proxy for disease score. Secondary lesions in all immunocompromised mice caused no discernable distress to the animals. All mice received constant pain medication (1g/L Dalfalgan diluted in drinking water) during the second half of their infection. However, swelling in the primary footpad lesions in LRV1+ *L*.*g* infected immunocompromised mice, posed an ethical limitation of 7 weeks. LRV1- *L*.*g* infected immunocompromised mice reached this ethical limit approximately 3–4 weeks later.

### Parasite quantification by luminescence

At the peak of infection (week 4/5) and at the onset of visible infectious metastasis (week 7/8), parasite burden was quantified using *in vivo* imaging able to detect luminescence emitted by luciferase-transfected parasites. Briefly, mice were injected intra-peritoneally with D-Luciferin sodium salt (Regis technologies) prepared in PBS at a final concentration of 150 mg/kg. After a 10 min incubation, mice were anesthetized by continuous gas anaesthesia and imaged using a Xenogen Lumina II imaging system (IVIS, 10 min exposure time). Total photon flux over the primary lesions or length of the tail was assessed using the associated software (Living Image).

### Parasite isolation and culture from metastatic lesions

Biopsies were taken from visible metastases in the tails, forepaws and snouts of affected mice 7 weeks post infection. Biopsy material was gently homogenized using a sterile tissue culture pestle and mortar. The lesion homogenate was then used to inoculate 3 culture dishes of complete Schneider’s insect medium. The presence of *Leishmania* parasites was confirmed by microscopy after 5 days of culture.

### Digoxin and SR1001 *in vivo* treatment protocol

Digoxin (Carbosynth) was diluted in PBS and SR1001 (Sigma-Aldrich) was dissolved in 100% DMSO before being diluted in PBS as previously described [37]. The final concentration of DMSO was 1.5%, which was used in the preparation of the vehicle control. At the onset of visibly swollen lesions (~2 weeks post inoculation), mice were injected intra-peritoneally every second day with 20 mg/kg (1250 μM) SR1001, or 40 μg/mouse of Digoxin, or vehicle control. Treatment continued for 3 weeks in wild type C57BL/6 mice (until week 5 of infection) and for 5–6 weeks in IFN-γ^-/-^ and IFN-γ^-/-^ IL-17A^-/-^ mice (until mice were humanely euthanized).

### Quantification of cytokine secretion in draining LNs

At the same time points as parasite quantification, lymphocytes were isolated from the LNs draining the primary lesions (popliteal) and those draining the metastatic tail lesions (iliac). Cells (5x10^6^/ml) were stimulated with UV-irradiated *L*.*g* LRV1+ and LRV1- promastigotes (1x10^6^/ml) in complete medium: DMEM (Gibco), supplemented with 10% heat-inactivated foetal bovine serum (FBS), 1% penicillin/streptomycin and 1% HEPES (Sigma-Aldrich). After 72 hours, various cytokines (IFN-γ, IL-17A, IL-17F and IL-17A/F) were quantified in cell-free culture supernatants by ELISA (eBioscience) following the manufacturer’s protocol.

### Flow cytometry analysis of draining lymph nodes

Lymphocytes isolated from draining LNs (described above) were treated with 50 ng/ml PMA and 3 μg/ml ionomycin in the presence of Brefeldin-A (10 μg/ml, Sigma) for 4 h at 37°C. Cells were prepared for staining with fluorescent antibodies by blocking non-specific antibody binding sites using an FcR blocking reagent (Miltenyi Biotec). Extracellular antigens were then stained with fluorescently labelled antibodies against CD3, CD4, CD8, CD19, CD49, TCRαβ and TCRγδ according to manufacturer’s instruction (eBioscience). Cells were then prepared for intracellular staining using an intracellular fixation and permeabilization buffer set (eBioscience) before adding intracellular antibodies against IL-17A and IFN-γ according to manufacturer’s instruction (eBioscience). Data was acquired on a FACSCalibur flow cytometer (BectonDickinson) and analyzed with FlowJo software (TreeStar).

### Primer sequences

FAM-MGB-labelled primer/probe sets for were designed with Primer Express software from Applied Biosystems.

**KMP11:**               Fwd: 5’-GAGCACACGGAGAAGATCAAC A-3’;

                              Rev: 5’-CAAGCAGCTCAGCGAACTTG-3’;

                              MGB probe: 5’-FAM-CTCGGAGCACTTCAA-3’;

**LRV1:**                   Fwd: 5’-GAGTGGGAGTCCCCCACAT-3’;

                              Rev: 5’-TGGATACAACCAGACGATTGCT-3’;

                              MGB probe: 5’-FAM CATTTATGTAGTTCCT-3’

***il17a* (human):**     Hs00174383-m1

***ifng* (human):**       Hs00174143-m1

***actin* (human)**:     Hs 99999903-m1

The parameters for 7300 Real Time System were set to 10min at 95°C followed by 40 cycles (15 sec at 95°C and 1 min at 60°C).

### Statistics

Statistical significance was determined using an unpaired parametric *t*-test (for single-point analysis on bar-graphs) or a one-way Anova (x/y disease curves) as calculated by GraphPad software (Prism v5). Significance was recognized when p ≤ 0.05 and represented in three ranks namely *: p ≤ 0.05, **: p ≤ 0.01 and ***: p ≤ 0.001.

### Ethics statement

#### Human sample collection

Human samples were extracted from 78 adult patients enrolled into the study following written informed consent at the Centre Hospitalier Andrée Rosemon in Cayenne, French Guiana following the hospital regulations (http://www.ch-cayenne.net/Droits-et-Devoirs.html). Ethical approval for this study was granted on the authority of the “Comité consultatif sur le traitement de l'information en matière de recherche” (DR-2011-291, 911217).

#### Animal experimentation

All animal protocols in this publication were approved by the Swiss Federal Veterinary Office (SFVO), under the authorization numbers 2113.1 and 2113.2. Animal handling and experimental procedures were undertaken with strict adherence to ethical guidelines set out by the SFVO and under inspection by the Department of Security and Environment of the State of Vaud, Switzerland. All mouse genotypes holding the risk for developing metastatic cutaneous lesions on the tail were treated prophylactically with pain medication (1g/L Dalfalgan diluted in drinking water) and showed no signs of discomfort under these conditions.

## Supporting Information

S1 Fig Production of IL17-A by PBMCs in patients infected with LRV1+ and LRV1- *L*. *guyanensis*. PBMCs (10^6^/ml) from subjects infected with LRV1+ (n = 6) or LRV1- (n = 6) *L*.*g* were restimulated *ex vivo* using live LRV1+*L*.*g* promastigotes (10^6^/ml). After 5 days, IL-17A production was analyzed by ELISA in the culture supernatant. Data are mean +/- SEM using at least 2 technical replicates per condition. Significance tested by an unpaired, parametric *t*-test and indicated as *: P<0.05, **P<0.005, ***P<0.0001
(TIF)

S2 Fig LRV1-dependent IL-17A secretion is produced by a variety of cell types. WT C57BL/6 mice were infected in the hind footpads with either LRV1+ or LRV1-*L*.*g* stationary-phase promastigotes. At the peak of infection (4 weeks), lymphocytes were extracted from popliteal LNs and prepared for intracellular cytokine flow cytometry. Briefly, cells were re-stimulated *ex vivo* with PMA/ionomycin in the presence of brefeldin-A. Extracellular antigens were then stained to mark the following cell types: T cells (CD3+), as well as their major lineages (CD4+ and CD8+) and receptor subtypes (TCRαβ and TCRγδ); B cells were marked using anti-CD19, while anti-CD49b was used as a pan-NK cell marker. Stained cells were fixed and permeabilized in preparation for intracellular staining of IL-17A and IFN-γ. The upper panel **(A to F)** shows pLN cells from LRV1+*L*.*g* infected WT mice while the lower panel **(G to L)** shows cells from LRV1-*L*.*g* infected WT mice. **L** is a representative graph for the isotype control of intracellular IL-17A staining. Graphs are representative of a minimum of 3 independent experiments, using at least 5 mice per condition and a minimum of 1x10^5^ events per plot. Plots are representative of the gated populations indicated at the top of each graph. The position of each gate is indicated as a black square.
(TIF)

S3 Fig Metastatic score in IFN-γ ^-/-^ mice. Mice deficient in IFN-γ were infected in the hind footpads with LRV1+ or LRV1-*L*.*g* stationary-phase promastigotes. The number of secondary lesions in the tail were counted once a week from the onset of metastasis until reaching the ethical limit of the experiment. Graphs are representative of a minimum of 3 independent experiments, using at least 5 mice per condition and presented as mean ± SEM. Significance is tested by an unpaired, parametric *t*-test and indicated as *: P<0.05, **P<0.005, ***P<0.0001.
(TIF)

S4 Fig IL-17A inhibiting drugs (Digoxin and SR1001) reduce disease phenotype in C57BL/6 mice. **(A-E)** IL-17A^-/-^ and their WT controls (C57BL/6) were infected in the hind footpads with LRV1+ *L*.*g* stationary-phase promastigotes. At the onset of visible disease (2 weeks post inoculation) mice were treated with Digoxin (40 μg/mouse prepared in PBS) or SR1001 (dissolved in 1.5% DMSO and PBS to a final concentration of 20 mg/kg) by intra-peritoneal injections every second day for 3 weeks. Footpad swelling was measured weekly as a proxy for disease progression in **(A)** WT and **(D)** IL-17A^-/-^ mice. Significance is depicted for comparisons between SR1001-treated or Digoxin treated mice and control groups. At the end of the treatment (week 5) *in vivo* parasite luminescence was determined as a measure of parasite burden in **(B)** WT and **(E)** IL-17A^-/-^ mice. **(C)** At week 7 post infection, lymphocytes were exacted from popliteal LNs of WT mice infected with LRV1+ *L*.*g* mice and then re-stimulated *ex vivo* with UV inactivated LRV1+ *L*.*g* parasites. IL-17A was then measured in cell-free supernatants by ELISA. **(F-G)** IL-17A^-/-^ IFN-γ^-/-^ mice were infected in the hind footpads with LRV1+ *L*.*g* stationary-phase promastigotes and treated from week 2.5 to week 7 with Digoxin or SR1001 as mentioned above. **(F)** Footpad swelling was monitored weekly as a proxy for disease progression. At the peak of infection (week 4), **(G)** parasite burden was quantified by *in vivo* parasite luminescence, after injecting mice intra-peritoneally with luciferin. **(H)** An enumeration of secondary metastatic lesions in the tail at week 7. Graphs are representative of a minimum of 3 independent experiments, using at least 5 mice per condition and presented as mean +/- SEM. Significance is tested by one-way Anova (disease score) or an unpaired, parametric *t*-test (bar graph) and indicated as *: P<0.05, **P<0.005, ***P<0.0001.
(TIF)

S5 Fig Absence of TCRγδ T cells does not impair the course of infection. Mice deficient in TCRδ and their WT controls (C57BL/6) were infected in the hind footpads with 3x10^6^ of either LRV1+ or LRV1-*L*.*g* stationary-phase promastigotes. **(A)** Change in footpad swelling in TCRγδ and WT mice was measured weekly as a proxy for disease score. **(B)** At the peak of infection, the parasite burden in these mice was quantified by *in vivo* parasite luminescence. Graphs are representative of a minimum of 3 independent experiments, using at least 5 mice per condition and presented as mean ± SEM. Significance tested by an unpaired, parametric *t*-test (bar graphs) or one-way Anova (disease score), and indicated as *: P<0.05, **P<0.005, ***P<0.0001.
(TIF)

## References

[1] World Health Organization. Control of the leishmaniases. World Health Organ Tech Rep Ser. 2010(949). 21485694

[2] Fischer D, Moeller P, Thomas SM, Naucke TJ, Beierkuhnlein C. Combining climatic projections and dispersal ability: a method for estimating the responses of sandfly vector species to climate change. PLoS Negl Trop Dis. 2011;5(11):e1407 10.1371/journal.pntd.0001407 22140590 PMC3226457

[3] Ready PD. Leishmaniasis emergence in Europe. Euro Surveill. 2010;15(10):19505 20403308

[4] Gonzalez C, Wang O, Strutz SE, Gonzalez-Salazar C, Sanchez-Cordero V, Sarkar S. Climate change and risk of leishmaniasis in north america: predictions from ecological niche models of vector and reservoir species. PLoS Negl Trop Dis. 2010;4(1):e585 10.1371/journal.pntd.0000585 20098495 PMC2799657

[5] Hartley MA, Drexler S, Ronet C, Beverley SM, Fasel N. The immunological, environmental, and phylogenetic perpetrators of metastatic leishmaniasis. Trends Parasitol. 2014; 10.1016/j.pt.2014.05.006 PMC4287268 24954794

[6] Goto H, Lauletta Lindoso JA. Cutaneous and mucocutaneous leishmaniasis. Infect Dis Clin North Am. 2012;26(2):293–307. 10.1016/j.idc.2012.03.001 22632640

[7] Alvar J, Velez ID, Bern C, Herrero M, Desjeux P, Cano J, et al Leishmaniasis worldwide and global estimates of its incidence. PLoS One. 2012;7(5):e35671 10.1371/journal.pone.0035671 22693548 PMC3365071

[8] Stuart KD, Weeks R, Guilbride L, Myler PJ. Molecular organization of Leishmania RNA virus 1. Proc Natl Acad Sci U S A. 1992;89(18):8596–600. 1382295 10.1073/pnas.89.18.8596 PMC49967

[9] Tarr PI, Aline RF Jr., Smiley BL, Scholler J, Keithly J, Stuart K. LR1: a candidate RNA virus of Leishmania. Proc Natl Acad Sci U S A. 1988;85(24):9572–5. 3200841 10.1073/pnas.85.24.9572 PMC282800

[10] Zangger H, Hailu A, Desponds C, Lye LF, Akopyants NS, Dobson DE, et al Leishmania aethiopica field isolates bearing an endosymbiontic dsRNA virus induce pro-inflammatory cytokine response. PLoS Negl Trop Dis. 2014;8(4):e2836 10.1371/journal.pntd.0002836 24762979 PMC3998932

[11] Zangger H, Ronet C, Desponds C, Kuhlmann FM, Robinson J, Hartley MA, et al Detection of Leishmania RNA virus in Leishmania parasites. PLoS Negl Trop Dis. 2013;7(1):e2006 10.1371/journal.pntd.0002006 23326619 PMC3542153

[12] Ives A, Ronet C, Prevel F, Ruzzante G, Fuertes-Marraco S, Schutz F, et al Leishmania RNA virus controls the severity of mucocutaneous leishmaniasis. Science. 2011;331(6018):775–8. 10.1126/science.1199326 21311023 PMC3253482

[13] Bourreau E, Ginouves M, Prevot G, Hartley MA, Gangneux JP, Robert-Gangneux F, et al Presence of Leishmania RNA Virus 1 in Leishmania guyanensis Increases the Risk of First-Line Treatment Failure and Symptomatic Relapse. J Infect Dis. 2016;213(1):105–11. Epub 2015/07/01. 10.1093/infdis/jiv355 26123564

[14] Adaui V, Lye LF, Akopyants NS, Zimic M, Llanos-Cuentas A, Garcia L, et al Association of the Endobiont Double-Stranded RNA Virus LRV1 With Treatment Failure for Human Leishmaniasis Caused by Leishmania braziliensis in Peru and Bolivia. J Infect Dis. 2016;213(1):112–21. Epub 2015/07/01. 10.1093/infdis/jiv354 26123565 PMC4676543

[15] Cantanhede LM, da Silva Junior CF, Ito MM, Felipin KP, Nicolete R, Salcedo JM, et al Further Evidence of an Association between the Presence of Leishmania RNA Virus 1 and the Mucosal Manifestations in Tegumentary Leishmaniasis Patients. PLoS Negl Trop Dis. 2015;9(9):e0004079 Epub 2015/09/16. 10.1371/journal.pntd.0004079 26372217 PMC4570810

[16] Ives A, Masina S, Castiglioni P, Prevel F, Revaz-Breton M, Hartley MA, et al MyD88 and TLR9 Dependent Immune Responses Mediate Resistance to Leishmania guyanensis Infections, Irrespective of Leishmania RNA Virus Burden. PLoS One. 2014;9(5):e96766 10.1371/journal.pone.0096766 24801628 PMC4011865

[17] Locksley RM, Heinzel FP, Holaday BJ, Mutha SS, Reiner SL, Sadick MD. Induction of Th1 and Th2 CD4+ subsets during murine Leishmania major infection. Res Immunol. 1991;142(1):28–32. 1829257 10.1016/0923-2494(91)90007-6

[18] Louis JA, Conceicao-Silva F, Himmelrich H, Tacchini-Cottier F, Launois P. Anti-leishmania effector functions of CD4+ Th1 cells and early events instructing Th2 cell development and susceptibility to Leishmania major in BALB/c mice. Adv Exp Med Biol. 1998;452:53–60. 9889959 10.1007/978-1-4615-5355-7_7

[19] Alexander J, Bryson K. T helper (h)1/Th2 and Leishmania: paradox rather than paradigm. Immunol Lett. 2005;99(1):17–23. 15894106 10.1016/j.imlet.2005.01.009

[20] Alexander J, Brombacher F. T helper1/t helper2 cells and resistance/susceptibility to leishmania infection: is this paradigm still relevant? Front Immunol. 2012;3:80 10.3389/fimmu.2012.00080 22566961 PMC3342373

[21] Hartley MA, Ronet C, Zangger H, Beverley SM, Fasel N. Leishmania RNA virus: when the host pays the toll. Front Cell Infect Microbiol. 2012;2:99 10.3389/fcimb.2012.00099 22919688 PMC3417650

[22] Tacchini-Cottier F, Weinkopff T, Launois P. Does T Helper Differentiation Correlate with Resistance or Susceptibility to Infection with L. major? Some Insights From the Murine Model. Front Immunol. 2012;3:32 10.3389/fimmu.2012.00032 22566916 PMC3342012

[23] Bedoya SK, Lam B, Lau K, Larkin J, 3rd. Th17 cells in immunity and autoimmunity. Clin Dev Immunol. 2013;2013:986789 10.1155/2013/986789 24454481 PMC3886602

[24] Yang B, Kang H, Fung A, Zhao H, Wang T, Ma D. The Role of Interleukin 17 in Tumour Proliferation, Angiogenesis, and Metastasis. Mediators Inflamm. 2014;2014:623759 10.1155/2014/623759 25110397 PMC4119694

[25] Kim JW, Kim YK, Hwang JA, Yoon HK, Ko YH, Han C, et al Plasma Levels of IL-23 and IL-17 before and after Antidepressant Treatment in Patients with Major Depressive Disorder. Psychiatry Investig. 2013;10(3):294–9. 10.4306/pi.2013.10.3.294 24302954 PMC3843023

[26] Holm CK, Petersen CC, Hvid M, Petersen L, Paludan SR, Deleuran B, et al TLR3 ligand polyinosinic:polycytidylic acid induces IL-17A and IL-21 synthesis in human Th cells. J Immunol. 2009;183(7):4422–31. 10.4049/jimmunol.0804318 19748983

[27] Lee SY, Yoon BY, Kim JI, Heo YM, Woo YJ, Park SH, et al Interleukin-17 increases the expression of Toll-like receptor 3 via the STAT3 pathway in rheumatoid arthritis fibroblast-like synoviocytes. Immunology. 2014;141(3):353–61. 10.1111/imm.12196 24708416 PMC3930374

[28] Hsia BJ, Whitehead GS, Thomas SY, Nakano K, Gowdy KM, Aloor JJ, et al Trif-dependent induction of Th17 immunity by lung dendritic cells. Mucosal Immunol. 2014; 10.1038/mi.2014.56 PMC4267961 24985082

[29] Said A, Bock S, Muller G, Weindl G. Inflammatory conditions distinctively alter immunological functions of Langerhans-like cells and dendritic cells in vitro. Immunology. 2014; 10.1111/imm.12363 PMC4298416 25059418

[30] He J, Lang G, Ding S, Li L. Pathological role of interleukin-17 in poly I:C-induced hepatitis. PLoS One. 2013;8(9):e73909 10.1371/journal.pone.0073909 24069246 PMC3777971

[31] Harrington LE, Hatton RD, Mangan PR, Turner H, Murphy TL, Murphy KM, et al Interleukin 17-producing CD4+ effector T cells develop via a lineage distinct from the T helper type 1 and 2 lineages. Nat Immunol. 2005;6(11):1123–32. 16200070 10.1038/ni1254

[32] Irmler IM, Gajda M, Brauer R. Exacerbation of antigen-induced arthritis in IFN-gamma-deficient mice as a result of unrestricted IL-17 response. J Immunol. 2007;179(9):6228–36. 17947698 10.4049/jimmunol.179.9.6228

[33] Kelchtermans H, Billiau A, Matthys P. How interferon-gamma keeps autoimmune diseases in check. Trends Immunol. 2008;29(10):479–86. 10.1016/j.it.2008.07.002 18775671

[34] Savarin C, Stohlman SA, Hinton DR, Ransohoff RM, Cua DJ, Bergmann CC. IFN-gamma protects from lethal IL-17 mediated viral encephalomyelitis independent of neutrophils. J Neuroinflammation. 2012;9:104 10.1186/1742-2094-9-104 22642802 PMC3419086

[35] Huh JR, Leung MW, Huang P, Ryan DA, Krout MR, Malapaka RR, et al Digoxin and its derivatives suppress TH17 cell differentiation by antagonizing RORgammat activity. Nature. 2011;472(7344):486–90. Epub 2011/03/29. 10.1038/nature09978 21441909 PMC3172133

[36] Coleman DT, Gray AL, Stephens CA, Scott ML, Cardelli JA. Repurposed drug screen identifies cardiac glycosides as inhibitors of TGF-beta-induced cancer-associated fibroblast differentiation. Oncotarget. 2016; 10.18632/oncotarget.8609. Epub 2016/04/09. PMC5078007 27058757

[37] Solt LA, Kumar N, Nuhant P, Wang Y, Lauer JL, Liu J, et al Suppression of TH17 differentiation and autoimmunity by a synthetic ROR ligand. Nature. 2011;472(7344):491–4. 10.1038/nature10075 21499262 PMC3148894

[38] Parmentier L, Cusini A, Muller N, Zangger H, Hartley MA, Desponds C, et al Severe Cutaneous Leishmaniasis in a Human Immunodeficiency Virus Patient Coinfected with Leishmania braziliensis and Its Endosymbiotic Virus. Am J Trop Med Hyg. 2016; 10.4269/ajtmh.15-0803. Epub 2016/02/03. PMC4824227 26834198

[39] de Oliveira Ramos Pereira L, Maretti-Mira AC, Rodrigues KM, Lima RB, de Oliveira-Neto MP, Cupolillo E, et al Severity of tegumentary leishmaniasis is not exclusively associated with Leishmania RNA virus 1 infection in Brazil. Mem Inst Oswaldo Cruz. 2013;108(5):665–7. 23903986 10.1590/0074-0276108052013021 PMC3970596

[40] Ginouves M, Simon S, Bourreau E, Lacoste V, Ronet C, Couppie P, et al Prevalence and Distribution of Leishmania RNA Virus 1 in Leishmania Parasites from French Guiana. Am J Trop Med Hyg. 2016;94(1):102–6. Epub 2015/11/26. 10.4269/ajtmh.15-0419 26598572 PMC4710412

[41] Pereira Lde O, Maretti-Mira AC, Rodrigues KM, Lima RB, Oliveira-Neto MP, Cupolillo E, et al Severity of tegumentary leishmaniasis is not exclusively associated with Leishmania RNA virus 1 infection in Brazil. Mem Inst Oswaldo Cruz. 2013;108(5):665–7. Epub 2013/08/02. 23903986 10.1590/0074-0276108052013021 PMC3970596

[42] O'Neal SE, Guimaraes LH, Machado PR, Alcantara L, Morgan DJ, Passos S, et al Influence of helminth infections on the clinical course of and immune response to Leishmania braziliensis cutaneous leishmaniasis. J Infect Dis. 2007;195(1):142–8. Epub 2006/12/08. 17152018 10.1086/509808

[43] Crosby EJ, Clark M, Novais FO, Wherry EJ, Scott P. Lymphocytic Choriomeningitis Virus Expands a Population of NKG2D+CD8+ T Cells That Exacerbates Disease in Mice Coinfected with Leishmania major. J Immunol. 2015;195(7):3301–10. Epub 2015/08/21. 10.4049/jimmunol.1500855 26290604 PMC4575880

[44] Swihart K, Fruth U, Messmer N, Hug K, Behin R, Huang S, et al Mice from a genetically resistant background lacking the interferon gamma receptor are susceptible to infection with Leishmania major but mount a polarized T helper cell 1-type CD4+ T cell response. J Exp Med. 1995;181(3):961–71. 7869054 10.1084/jem.181.3.961 PMC2191906

[45] Pinheiro RO, Rossi-Bergmann B. Interferon-gamma is required for the late but not early control of Leishmania amazonensis infection in C57Bl/6 mice. Mem Inst Oswaldo Cruz. 2007;102(1):79–82. 17294004 10.1590/s0074-02762007000100013

[46] Bacellar O, Lessa H, Schriefer A, Machado P, Ribeiro de Jesus A, Dutra WO, et al Up-regulation of Th1-type responses in mucosal leishmaniasis patients. Infect Immun. 2002;70(12):6734–40. 12438348 10.1128/IAI.70.12.6734-6740.2002 PMC132996

[47] Hartley MA, Kohl K, Ronet C, Fasel N. The therapeutic potential of immune cross-talk in leishmaniasis. Clin Microbiol Infect. 2013;19(2):119–30. 10.1111/1469-0691.12095 23398405

[48] Lopez Kostka S, Dinges S, Griewank K, Iwakura Y, Udey MC, von Stebut E. IL-17 promotes progression of cutaneous leishmaniasis in susceptible mice. J Immunol. 2009;182(5):3039–46. 10.4049/jimmunol.0713598 19234200 PMC2658650

[49] Gonzalez-Lombana C, Gimblet C, Bacellar O, Oliveira WW, Passos S, Carvalho LP, et al IL-17 mediates immunopathology in the absence of IL-10 following Leishmania major infection. PLoS Pathog. 2013;9(3):e1003243 10.1371/journal.ppat.1003243 23555256 PMC3605236

[50] Boaventura VS, Santos CS, Cardoso CR, de Andrade J, Dos Santos WL, Clarencio J, et al Human mucosal leishmaniasis: neutrophils infiltrate areas of tissue damage that express high levels of Th17-related cytokines. Eur J Immunol. 2010;40(10):2830–6. 10.1002/eji.200940115 20812234

[51] Xin L, Li Y, Soong L. Role of interleukin-1beta in activating the CD11c(high) CD45RB- dendritic cell subset and priming Leishmania amazonensis-specific CD4+ T cells in vitro and in vivo. Infect Immun. 2007;75(10):5018–26. 17682041 10.1128/IAI.00499-07 PMC2044509

[52] Vargas-Inchaustegui DA, Xin L, Soong L. Leishmania braziliensis infection induces dendritic cell activation, ISG15 transcription, and the generation of protective immune responses. J Immunol. 2008;180(11):7537–45. 18490754 10.4049/jimmunol.180.11.7537 PMC2641013

[53] Bourreau E, Ronet C, Darcissac E, Lise MC, Sainte Marie D, Clity E, et al Intralesional regulatory T-cell suppressive function during human acute and chronic cutaneous leishmaniasis due to Leishmania guyanensis. Infect Immun. 2009;77(4):1465–74. 10.1128/IAI.01398-08 19168733 PMC2663152

[54] Wortmann GW, Aronson NE, Miller RS, Blazes D, Oster CN. Cutaneous leishmaniasis following local trauma: a clinical pearl. Clin Infect Dis. 2000;31(1):199–201. 10913426 10.1086/313924

[55] Bertho AL, Santiago MA, Coutinho SG. An experimental model of the production of metastases in murine cutaneous leishmaniasis. J Parasitol. 1994;80(1):93–9. 8308664

[56] Scheffter SM, Ro YT, Chung IK, Patterson JL. The complete sequence of Leishmania RNA virus LRV2-1, a virus of an Old World parasite strain. Virology. 1995;212(1):84–90. 7676652 10.1006/viro.1995.1456

[57] O'Connell RM, Taganov KD, Boldin MP, Cheng G, Baltimore D. MicroRNA-155 is induced during the macrophage inflammatory response. Proc Natl Acad Sci U S A. 2007;104(5):1604–9. 17242365 10.1073/pnas.0610731104 PMC1780072

[58] Escobar T, Yu CR, Muljo SA, Egwuagu CE. STAT3 activates miR-155 in Th17 cells and acts in concert to promote experimental autoimmune uveitis. Invest Ophthalmol Vis Sci. 2013;54(6):4017–25. 10.1167/iovs.13-11937 23674757 PMC3680004

[59] Yao R, Ma YL, Liang W, Li HH, Ma ZJ, Yu X, et al MicroRNA-155 modulates Treg and Th17 cells differentiation and Th17 cell function by targeting SOCS1. PLoS One. 2012;7(10):e46082 10.1371/journal.pone.0046082 23091595 PMC3473054

[60] Hu R, Huffaker TB, Kagele DA, Runtsch MC, Bake E, Chaudhuri AA, et al MicroRNA-155 confers encephalogenic potential to Th17 cells by promoting effector gene expression. J Immunol. 2013;190(12):5972–80. 10.4049/jimmunol.1300351 23686497 PMC3773482

[61] Gollob KJ, Antonelli LR, Faria DR, Keesen TS, Dutra WO. Immunoregulatory mechanisms and CD4-CD8- (double negative) T cell subpopulations in human cutaneous leishmaniasis: a balancing act between protection and pathology. Int Immunopharmacol. 2008;8(10):1338–43. 10.1016/j.intimp.2008.03.016 18687296 PMC3575027

[62] Bottrel RL, Dutra WO, Martins FA, Gontijo B, Carvalho E, Barral-Netto M, et al Flow cytometric determination of cellular sources and frequencies of key cytokine-producing lymphocytes directed against recombinant LACK and soluble Leishmania antigen in human cutaneous leishmaniasis. Infect Immun. 2001;69(5):3232–9. 11292745 10.1128/IAI.69.5.3232-3239.2001 PMC98281

[63] Wang HX, Chu S, Li J, Lai WN, Wang HX, Wu XJ, et al Increased IL-17 and IL-21 producing TCRalphabeta+CD4-CD8- T cells in Chinese systemic lupus erythematosus patients. Lupus. 2014;23(7):643–54. 10.1177/0961203314524467 24554709

[64] Rotureau B, Ravel C, Couppie P, Pratlong F, Nacher M, Dedet JP, et al Use of PCR-restriction fragment length polymorphism analysis to identify the main new world Leishmania species and analyze their taxonomic properties and polymorphism by application of the assay to clinical samples. J Clin Microbiol. 2006;44(2):459–67. Epub 2006/02/04. 16455899 10.1128/JCM.44.2.459-467.2006 PMC1392689

[65] Lye LF, Owens K, Shi H, Murta SM, Vieira AC, Turco SJ, et al Retention and loss of RNA interference pathways in trypanosomatid protozoans. PLoS Pathog. 2010;6(10):e1001161 10.1371/journal.ppat.1001161 21060810 PMC2965760

[66] Ro YT, Scheffter SM, Patterson JL. Hygromycin B resistance mediates elimination of Leishmania virus from persistently infected parasites. J Virol. 1997;71(12):8991–8. 9371555 10.1128/jvi.71.12.8991-8998.1997 PMC230199

[67] Bourreau E, Prevot G, Gardon J, Pradinaud R, Launois P. High intralesional interleukin-10 messenger RNA expression in localized cutaneous leishmaniasis is associated with unresponsiveness to treatment. J Infect Dis. 2001;184(12):1628–30. 11740743 10.1086/324665

[68] Nakae S, Komiyama Y, Nambu A, Sudo K, Iwase M, Homma I, et al Antigen-specific T cell sensitization is impaired in IL-17-deficient mice, causing suppression of allergic cellular and humoral responses. Immunity. 2002;17(3):375–87. 12354389 10.1016/s1074-7613(02)00391-6

