# Supplementary figures and images for: Leishmaniavirus-Dependent Metastatic Leishmaniasis Is Prevented by Blocking IL-17A
Source: PLoS Pathog. 2016 Sep 22;12(9):e1005852. doi: 10.1371/journal.ppat.1005852 (PMC5033371; doi:10.1371/journal.ppat.1005852)

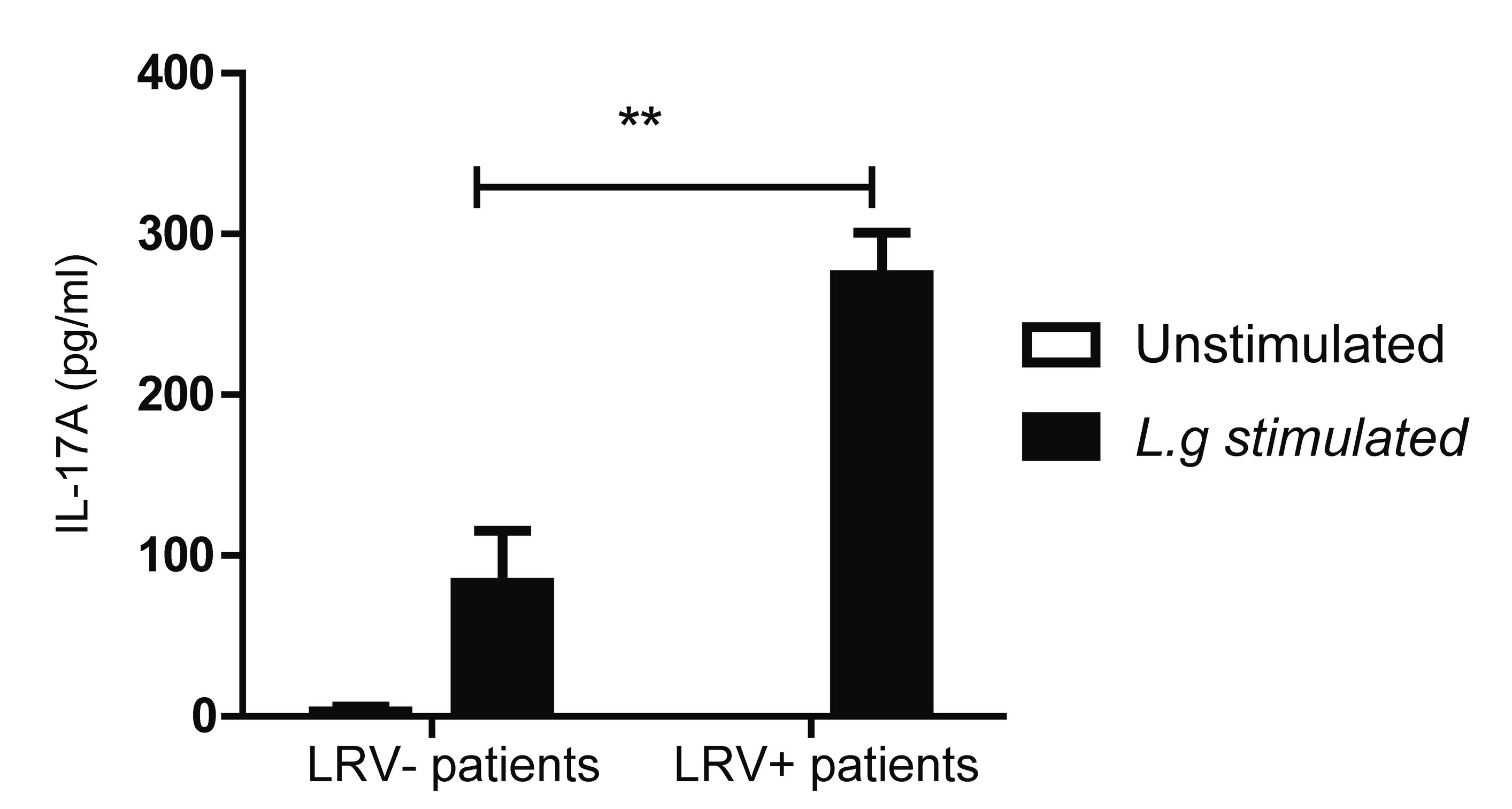

Supplement: S1 Fig — PBMCs (106/ml) from subjects infected with LRV1+ (n = 6) or LRV1- (n = 6) L.g were restimulated ex vivo using live LRV1+L.g promastigotes (106/ml). After 5 days, IL-17A production was analyzed by ELISA in the culture supernatant. Data are mean +/- SEM using at least 2 technical replicates per condition. Significance tested by an unpaired, parametric t-test and indicated as *: P<0.05, **P<0.005, ***P<0.0001 (TIF) [file ppat.1005852.s001.tif]

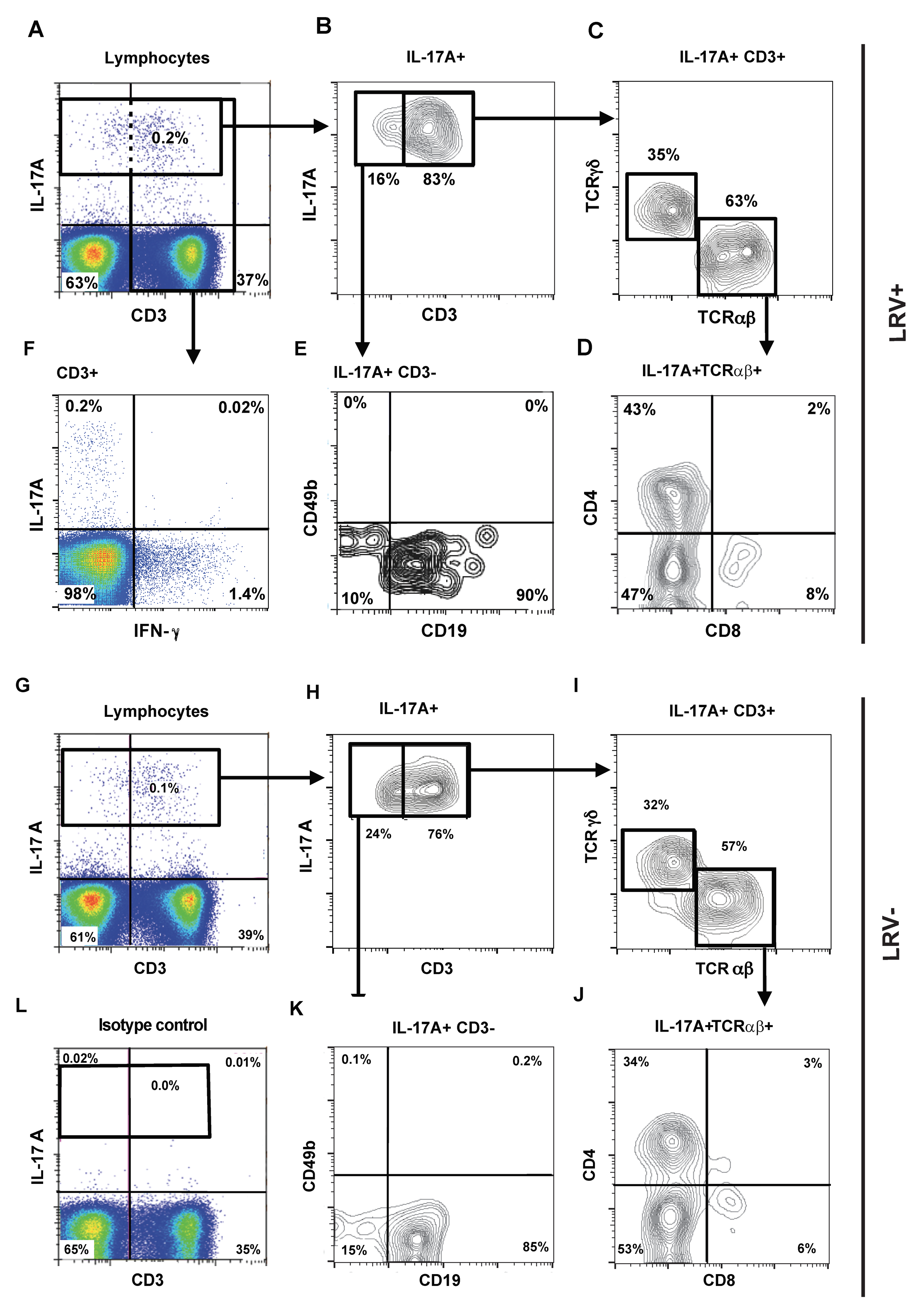

Supplement: S2 Fig — WT C57BL/6 mice were infected in the hind footpads with either LRV1+ or LRV1-L.g stationary-phase promastigotes. At the peak of infection (4 weeks), lymphocytes were extracted from popliteal LNs and prepared for intracellular cytokine flow cytometry. Briefly, cells were re-stimulated ex vivo with PMA/ionomycin in the presence of brefeldin-A. Extracellular antigens were then stained to mark the following cell types: T cells (CD3+), as well as their major lineages (CD4+ and CD8+) and receptor subtypes (TCRαβ and TCRγδ); B cells were marked using anti-CD19, while anti-CD49b was used as a pan-NK cell marker. Stained cells were fixed and permeabilized in preparation for intracellular staining of IL-17A and IFN-γ. The upper panel (A to F) shows pLN cells from LRV1+L.g infected WT mice while the lower panel (G to L) shows cells from LRV1-L.g infected WT mice. L is a representative graph for the isotype control of intracellular IL-17A staining. Graphs are representative of a minimum of 3 independent experiments, using at least 5 mice per condition and a minimum of 1x10^5^ events per plot. Plots are representative of the gated populations indicated at the top of each graph. The position of each gate is indicated as a black square. (TIF) [file ppat.1005852.s002.tif]

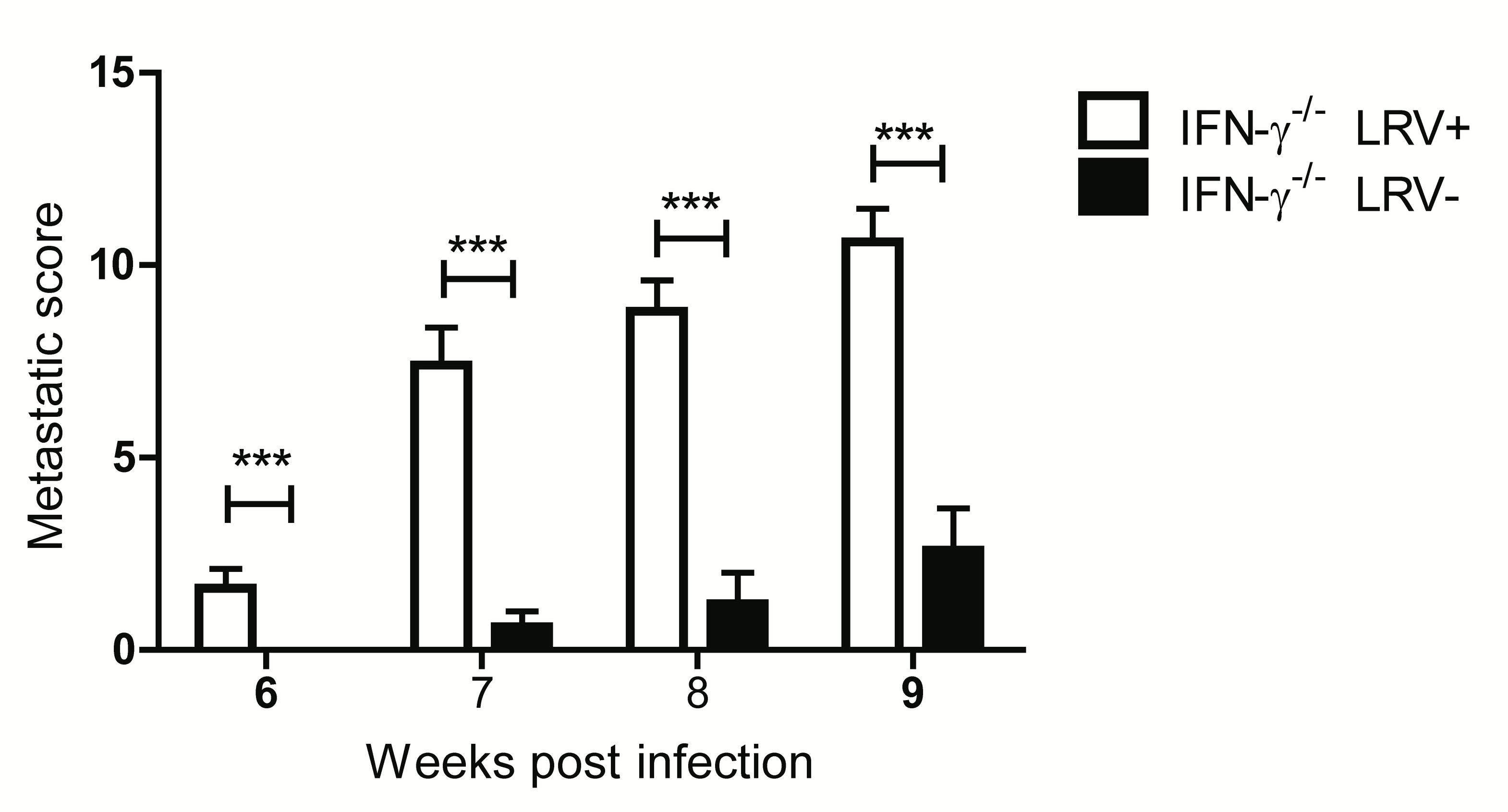

Supplement: S3 Fig — Mice deficient in IFN-γ were infected in the hind footpads with LRV1+ or LRV1-L.g stationary-phase promastigotes. The number of secondary lesions in the tail were counted once a week from the onset of metastasis until reaching the ethical limit of the experiment. Graphs are representative of a minimum of 3 independent experiments, using at least 5 mice per condition and presented as mean ± SEM. Significance is tested by an unpaired, parametric t-test and indicated as *: P<0.05, **P<0.005, ***P<0.0001. (TIF) [file ppat.1005852.s003.tif]

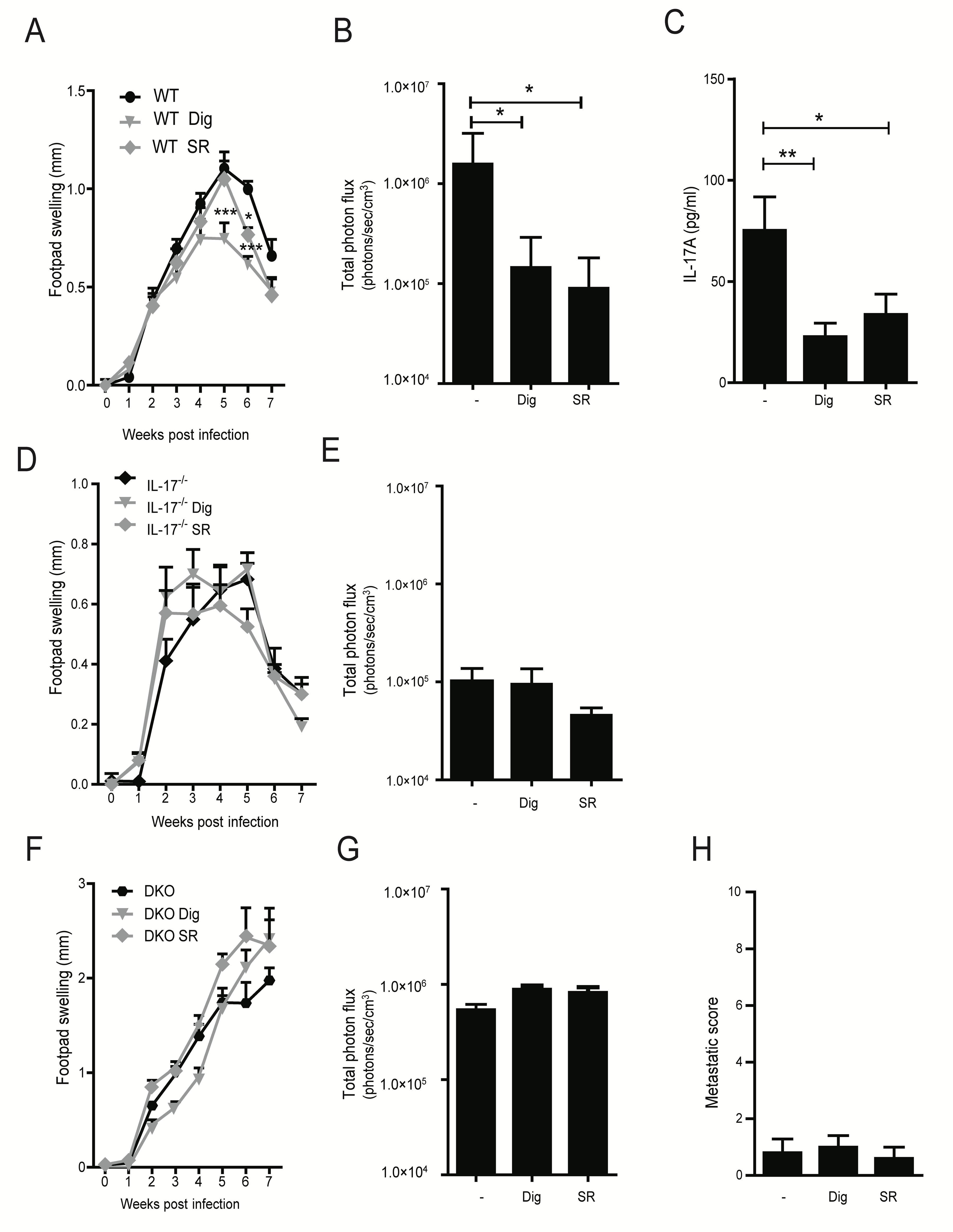

Supplement: S4 Fig — (A-E) IL-17A-/- and their WT controls (C57BL/6) were infected in the hind footpads with LRV1+ L.g stationary-phase promastigotes. At the onset of visible disease (2 weeks post inoculation) mice were treated with Digoxin (40 μg/mouse prepared in PBS) or SR1001 (dissolved in 1.5% DMSO and PBS to a final concentration of 20 mg/kg) by intra-peritoneal injections every second day for 3 weeks. Footpad swelling was measured weekly as a proxy for disease progression in (A) WT and (D) IL-17A-/- mice. Significance is depicted for comparisons between SR1001-treated or Digoxin treated mice and control groups. At the end of the treatment (week 5) in vivo parasite luminescence was determined as a measure of parasite burden in (B) WT and (E) IL-17A-/- mice. (C) At week 7 post infection, lymphocytes were exacted from popliteal LNs of WT mice infected with LRV1+ L.g mice and then re-stimulated ex vivo with UV inactivated LRV1+ L.g parasites. IL-17A was then measured in cell-free supernatants by ELISA. (F-G) IL-17A-/- IFN-γ-/- mice were infected in the hind footpads with LRV1+ L.g stationary-phase promastigotes and treated from week 2.5 to week 7 with Digoxin or SR1001 as mentioned above. (F) Footpad swelling was monitored weekly as a proxy for disease progression. At the peak of infection (week 4), (G) parasite burden was quantified by in vivo parasite luminescence, after injecting mice intra-peritoneally with luciferin. (H) An enumeration of secondary metastatic lesions in the tail at week 7. Graphs are representative of a minimum of 3 independent experiments, using at least 5 mice per condition and presented as mean +/- SEM. Significance is tested by one-way Anova (disease score) or an unpaired, parametric t-test (bar graph) and indicated as *: P<0.05, **P<0.005, ***P<0.0001. (TIF) [file ppat.1005852.s004.tif]

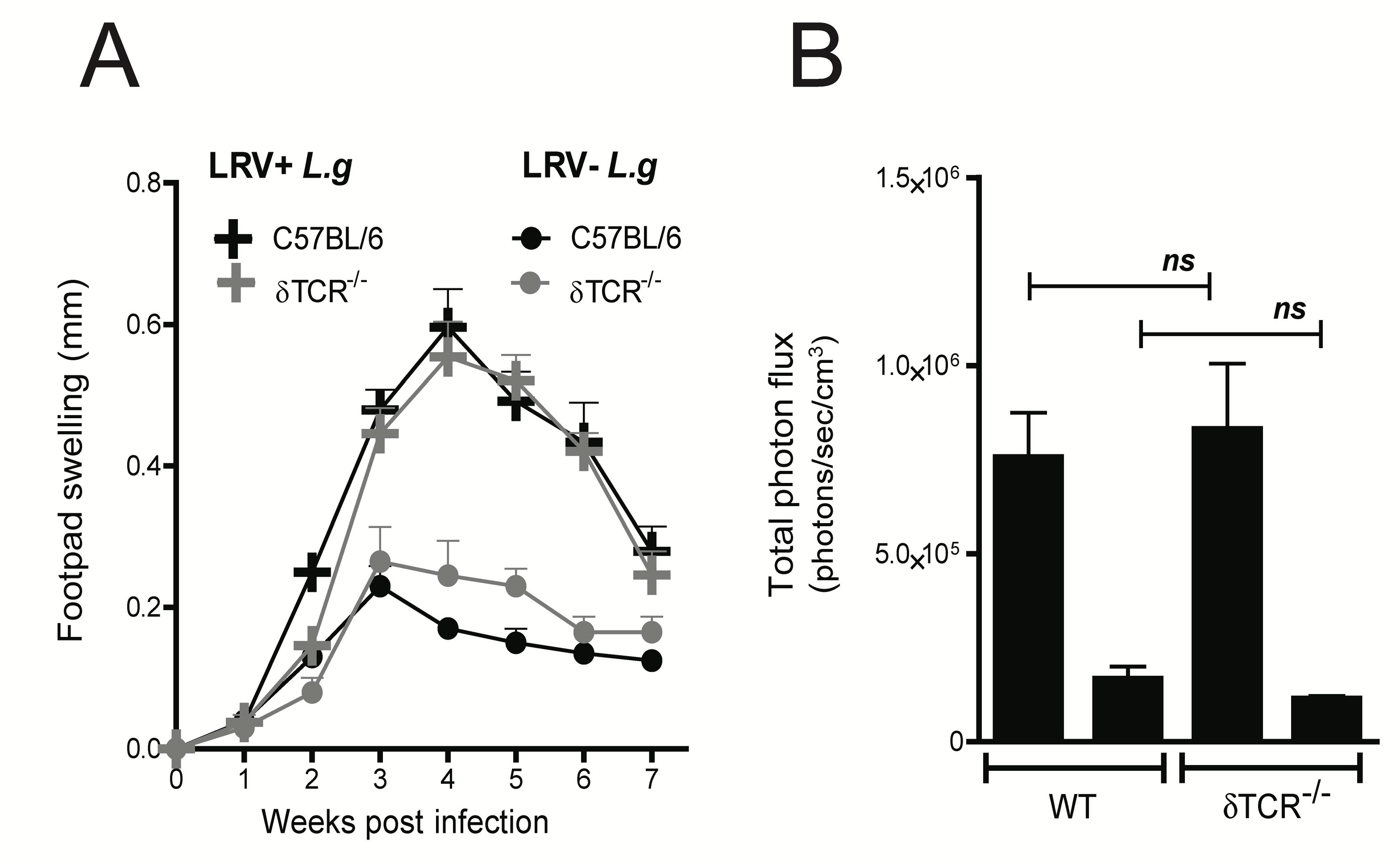

Supplement: S5 Fig — Mice deficient in TCRδ and their WT controls (C57BL/6) were infected in the hind footpads with 3x10^6^ of either LRV1+ or LRV1-L.g stationary-phase promastigotes. (A) Change in footpad swelling in TCRγδ and WT mice was measured weekly as a proxy for disease score. (B) At the peak of infection, the parasite burden in these mice was quantified by in vivo parasite luminescence. Graphs are representative of a minimum of 3 independent experiments, using at least 5 mice per condition and presented as mean ± SEM. Significance tested by an unpaired, parametric t-test (bar graphs) or one-way Anova (disease score), and indicated as *: P<0.05, **P<0.005, ***P<0.0001. (TIF) [file ppat.1005852.s005.tif]
